# The predictive role of aortic propagation velocity for coronary artery disease

**DOI:** 10.1186/s12872-018-0854-9

**Published:** 2018-06-19

**Authors:** Fereshteh Ghaderi, Hossein Samim, Faeze Keihanian, Seyed Ali Danesh Sani

**Affiliations:** 1 Fellowship in echocardiography, Cardiology Department, Imam Reza Hospital, Faculty of Medicine, Mashhad University of Medical Sciences, Mashhad, Iran; 2 Cardiology Resident, Cardiology Department, Faculty of Medicine, Imam Reza and Ghaem Hospitals, Mashhad University of Medical Sciences, Shariati Square, Mashhad, Iran; 3 Clinician Scientist of Cardiology, Pharmaceutical Research Division, Booali Research Center, Mashhad University of Medical Sciences, Mashhad, Iran; 4 General Practitioner, Cardiology department, Faculty of Medicine, Imam Reza Hospital, Mashhad University of Medical Sciences, Mashhad, Iran

**Keywords:** Arterial stiffness, Aortic propagation velocity, Echocardiography, Coronary artery disease

## Abstract

**Background:**

It is well recognized that cardio- vascular risk factors lead to histological and functional changes in aorta, and aortic stiffness is the best predictor of cardiovascular morbidity and mortality. In this study we evaluated the relation of a less evaluated echocardiographic parameter of aortic stiffness, aortic propagation velocity (APV) with the presence and severity of CAD.

**Methods:**

This cross sectional study was conducted from May 2015 to March 2016 in Imam Reza hospital, Mashhad, Iran. Seventy patients who were referred for elective coronary artery angiography were enrolled. Patients were divided into two sub-groups based on angiographic findings: patients with CAD (38 patients, 54.3%) and non-CAD (32 patients, 45.7%). Transthoracic echocardiography was performed using the conventional 2D and color M-Mode imaging. Aortic propagation velocity (APV), aortic strain (AS) and distensibility (AD) were measured. The presence and Severity of CAD (assessing by syntax score) and their relation with aortic stiffness indices were assessed.

**Results:**

Aortic strain (6.23 ± 1.93% versus 11.66 ± 4.86%, *P* < 0.0001), distensibility (2.46 ± 0.91 vs 5.57 ± 2.25 cm 2 dyn-110-3, *P* < 0.0001) and APV (48.63 ± 10.31 cm/sec vs 77.75 ± 9.97 cm/s, P < 0.0001) were significantly decreased in CAD group compared with non-CAD group. In our study, APV showed significant inverse relationship with CAD. Based on our results, APV less than 56 cm/sec could be used to predict CAD with sensitivity and specificity of 96.9 and 78.9% respectively. We also found an inverse correlation between APV and severity of CAD.

**Conclusion:**

Aortic strain, AD and APV (a less evaluated echocardiographic index) showed significant inverse correlation with presence and severity of CAD**.**

## Background

As cardiovascular diseases are the leading cause of mortality, the prevention of its complications is a major goal of healthcare. Interest is increasing in finding diagnosis of preclinical vascular involvement in cardiovascular disorders [1]. It is well known that aging and cardiovascular risk factors can alter structure and function of large arteries and their stiffness [2, 3]. Aortic stiffness is the best predictor of cardiovascular mortality and morbidity [4, 5]. Invasive assessment of arterial stiffness is not accessible in clinic and non-invasive options such as M-mode echocardiography has been recommended as a reproducible, accurate, simple and non-expensive technique [6]. Echocardiographic parameters such as aortic strain (AS), aortic distensibility (AD), pulse pressure, augmentation index pulse wave propagation velocity in the detection of aortic stiffness were suggested [7]. Previous studies were evaluated some of these parameters in different status such as atherosclerosis, hypertension, end stage renal disease, patients with chest pain and general population [8]. The literature on the relation between APV and CAD is limited. It is shown that APV in descending aorta in thoracic part is correlated with CAD with conversed pattern [7, 9]. Higher resistance in aortic wall due to atherosclerosis can indicate a decrement in flow propagation speed in the lumen of arteries [10]. While the extent of coronary artery disease (CAD) increased, the distension of aortic wall and its strain will be decreased. The relation between the presence of CAD severity and aortic strain is, also reported [11]. The aim of this study was to investigate the correlation of a less evaluated echocardiographic index of aortic stiffness (APV) with the presence and severity of CAD.

## Methods

### Subjects

**Fig. 1:**
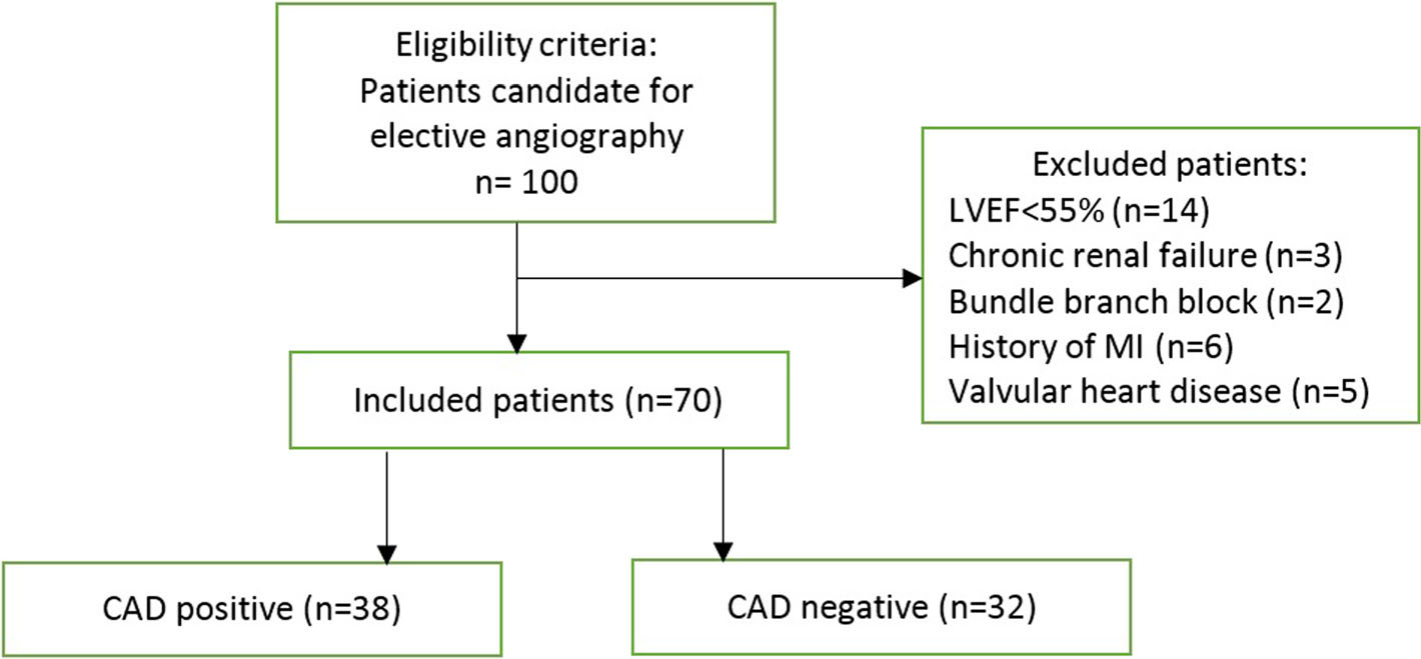
Flow diagram of study. CAD: coronary artery disease; LVEF: left ventricular ejection fraction; MI: myocardial infarction

The study group consisted of 100 consecutive patients who were referred for elective coronary angiography at our hospital 2015–2016 and divided into a CAD group and a non-CAD group (Fig. 1). A total of 30 patients excluded from the study because the following exclusion criteria: Significant valvular heart disease, known systemic disease affecting aorta (Marfan syndrome, etc.) aortic dilation more than 40 mm, chronic renal failure (CRF: eGFR< 60 ml/min/1.73m^2^), history of myocardial infarction, LVEF< 55%, arrhythmia (Every type of ventricular and atrial arrhythmia was excluded and none of participants during the study had APCs or VPCs), bundle branch block (Left and right bundle branch block), suboptimal suprasternal images and refusal of enrollment in the study.

### Sample size

We calculated sample size based on previous study [12] and below formula:

$$ \mathrm{n}=\frac{{\left({\mathrm{Z}}_{1-\raisebox{1ex}{$\upalpha $}\!\left/ \!\raisebox{-1ex}{$2$}\right.}+{\mathrm{Z}}_{1-\upbeta}\right)}^2\left({\mathrm{s}}_1^2+{\mathrm{s}}_2^2\right)}{\left({\overline{\mathrm{x}}}_1-{\overline{\mathrm{x}}}_2\right)2} $$

The residual 70 patients composed of 38 patients with obstructive coronary atherosclerosis in CAD group (who had more than 50% stenosis in at least one coronary artery) and 32 patients in non-CAD group who had normal coronary arteries or less than 50%coronary artery stenosis. In CAD group extent and severity of CAD was determined by Syntax score. We used SYNTAX score-I for determination of severity of CAD. All patients underwent TTE and APV, AS, AD were measured. An informed consent was given by all patients.

#### Coronary angiography

Coronary angiography was performed with Siemens Artis-zee (*Siemens* Healthcare, Forchheim, *Germany)* angiography devices. More than 50% stenotic lesions in left main or main three coronary arteries were considered as obstructive and the number of affected coronary arteries was determined. The severity of CAD was calculated by SYNTAX score I [13].

#### Transthoracic echocardiographic

Conventional and color M-mode echocardiography was performed using *Philips* iE33 (*Philips Healthcare,* Eindhoven, Netherlands*) with S 5 probe* by an experienced echocardiologist (first author) who was blinded to the angiographic data. M-mode Doppler tracing were recorded at the sweep rate of 200 mm/s. Three consecutive beats were averaged for every parameter. Ejection fraction, left ventricular end-systolic, and end-diastolic diameters, septal and posterior LV thickness were recorded.

##### Systolic and diastolic diameters of aorta measurement

The systolic and diastolic diameters of the ascending aorta were measured with M mode echocardiography 3 cm above the aortic annulus in the parasternal long axis view. The aortic systolic diameter was measured when the aortic valve was fully open and diastolic diameter at the peak of QRS complex on the simultaneously recorded electrocardiogram.

##### Aortic propagation velocity measurement

**Fig. 2:**
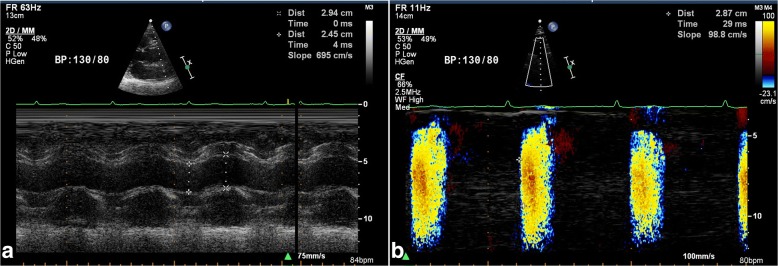
Measured (A) size of aorta and (B) aortic velocity propagation in a non-CAD case

Color M-mode Doppler tracing were obtained with the cursor parallel to the main flow in the lumen of descending aorta from the suprasternal window. The aliasing velocity was adjusted to 30–50 cm/s. A flame shaped color M-mode image was displayed by switching to M-mode tracing Aortic propagation velocity was measured by tracing the velocity slope of the first aliasing contour (Fig. 2). Systolic and diastolic blood pressure were recorded at the time of echocardiographic exam.

##### Aortic distensibility and aortic strain measurement

AS and AD were calculated based on the formula as below [14]:

$$ AS\left(\%\right)\frac{\left(\mathrm{aortic}\ \mathrm{systolic}\ \mathrm{diameter}-\mathrm{diastolic}\ \mathrm{diameter}\right)\times 100}{\mathrm{diastolic}\ \mathrm{diameter}.} $$

$$ AD\ \left({cm}^2/ dyn\right)=\frac{2\times \mathrm{AS}}{\mathrm{systolic}\ \mathrm{pressure}-\mathrm{diastolic}\ \mathrm{pressure}} $$

### Estimated glomerular filtration rate (eGFR) measurement

Estimated GFR (eGFR) was defined by MDRD formula:

$$ \mathrm{eGFR}\ \left(\mathrm{mL}/\min /1.73{\mathrm{m}}^2\right)=186\ {\left(\mathrm{S}.\mathrm{Cr}\ \mathrm{in}\ \upmu \mathrm{mol}/1\ \mathrm{x}\ 0.011312\right)}^{\hbox{-} 1.154}\mathrm{x}\ {\left(\mathrm{age}\right)}^{\hbox{-} 0.203}\ \mathrm{x}\ \left(0.742\ \mathrm{if}\ \mathrm{female}\right) $$

### Statistics

All data were entered in SPSS software (Statistical Package for Social Sciences for Windows, version 16.0, Chicago, IL, USA) and analyzed. All descriptive quantitative characteristics were reported as mean ± standard deviation and qualitative ones reported as percentage and counts. We analyzed normality of distribution for variables using Kolmogorov–Smirnov and Shapiro–Wilk tests. Pearson correlation and chi square tests were used for relationship of qualitative variables. Independent T-Test or One-way ANOVA were used for comparison of quantitative parameters for parametric variables and Mann Whitney Test for non-parametric ones. Multivariate regression analysis was used for predicting value of aortic stiffness parameters. The value of diagnosis of APV for CAD was evaluated with receiver operating characteristic (ROC) curve. *P*-Value lower than 0.05 was considered significant.

### Ethics

This study was approved by ethical committee of medical faculty of Mashhad University of Medical Sciences. Ethical code of the study was IR.MUMS.fm.REC.1394.141. All patients filled written informed consent form before entering the study.

## Results

Thirty patients were excluded from the analysis according to exclusion criteria, therefore the statistical analysis included 70 patients. As a result, 38 patients in the CAD group (54.3%) and 32 patients (45.7%) in the non-CAD group were included. The characteristics of the study population are shown in Table 1. There were no significant differences between the groups regarding age, gender, BMI and coronary risk factors including hypertension, hyperlipidemia diabetes mellitus, positive family history and smoking status.

**Table 1:** Comparison of clinical and demographic characteristics of patients in CAD or non-CAD groups

Characteristics	CAD group (*n* = 38)	Non-CAD group (*n* = 32)	Total (*n* = 70)	*P*-Value
Age (year) (mean ± S.D)	58.47 ± 13.29	58.12 ± 9.65	58.31 ± 11.69	0.20
Body Mass Index (Kg/m2)	26.28 ± 4.65	25.98 ± 4.38	26.12 ± 4.48	0.71
Height (cm)	163.37 ± 9.27	165.02 ± 9.57	164.27 ± 9.40	0.46
Weight (Kg)	70.06 ± 15.01	70.28 ± 13.22	70.18 ± 13.97	0.52
Heart rate (Per Minute)	71.62 ± 8.32	73.47 ± 12.17	72.62 ± 10.55	0.45
Systolic blood pressure (mmHg)	131.71 ± 14.95	143.02 ± 20.78	137.85 ± 19.08	0.10
Diastolic blood pressure (mmHg)	86.56 ± 12.40	89.86 ± 10.99	88.35 ± 11.69	0.24
Pulse Pressure (mmHg)	44.84 ± 14.05	53.68 ± 16.63	49.64 ± 16.02	0.21
Hypertension (N, %)	23, 60.5	18, 56.3	41, 58.6	0.71
Diabetes mellitus (N, %)	11, 28.9	10, 31.3	21, 30	0.83
Positive familial history (N, %)	13, 34.2	6, 18.8	19, 27.1	0.14
Smoking (N, %)	11, 28.9	10, 31.3	21, 30	0.87
Hyperlipidemia (N, %)	10, 26.3	8, 25	18, 25.7	0.90
SYNTAX score	12.46 ± 8.62	1.40 ± 4.11	7.40 ± 8.89	< 0.001
Left main disease (N, %)	2, 5.2	0	2, 100	–
Number of involved vessels (N, %)				
Normal coroner	0	32, 100	32, 100	
One-vessel disease	15, 39.4	0	15, 100	
Two-vessel disease	9, 23.6	0	9, 23.6	
Three-vessel disease	14, 36.8	0	14, 36.8	

Left ventricle end-diastolic and systolic dimension, LV thickness, aortic systolic and diastolic diameters, systolic and diastolic blood pressure, and pulse pressure were not statistically different between the groups. There were significantly difference in AS and AD between the groups (*P* < 0.001). (Table 2)

**Table 2:** echocardiographic findings in CAD and non-CAD groups

Characteristics	CAD group *n* = 38	Non-CAD group *n* = 32	*P*-Value
EF (%)	56.34 ± 2.79	56.56 ± 2.36	0.81
LVEDD (cm)	4.89 ± 0.32	4.76 ± 0.43	0.73
LVESD (cm)	3.00 ± 0.45	2.87 ± 0.40	0.23
PWT (cm)	0.80 ± 0.09	0.79 ± 0.08	0.48
ST (cm)	0.87 ± 0.12	0.83 ± 0.10	0.14
Diastolic diameter (cm)	2.93 ± 0.35	2.92 ± 0.23	0.69
Systolic diameter (cm)	3.14 ± 0.30	3.26 ± 0.24	0.76
AS (%)	6.23 ± 1.93	11.66 ± 4.86	< 0.0001
AD (cm ^2^dyn^−1^10^−3^)	2.46 ± 0.91	5.57 ± 2.25	< 0.0001
APV	48.63 ± 10.31	77.75 ± 9.97	< 0.0001

*EF* Ejection fraction; *LVEDD* Left ventricular end diastolic diameter; *LVESD* Left ventricular end systolic diameter; *PWT* Posterior wall thickness; *ST* Septal thickness; *AS* Aortic strain; *AD* Aortic distentibility; *APV* Aortic propagation velocity

APV was significantly lower in CAD rather than non-CAD group 77.75 ± 9.97 cm/sec (range: 55-97 cm/sec) versus 48.63 ± 10.31 cm/sec (range: 26-78 cm/sec, *P* < 0.0001) respectively. (Table 2).

There was positive correlation between APV with AS (*r* = 0.515, *P* < 0.0001) and AD (*r* = 0.599, *P* < 0.0001). There was significant reverse correlation between number of affected vessels and APV (*r* = − 0.791, P < 0.0001), AD (*r* = − 0.620, P < 0.0001) and AS (*r* = − 0.541, *P* < 0.0001). APV was significantly correlated with Syntax score (*r* = − 0.791, *P* < 0.0001).

**Fig. 3:**
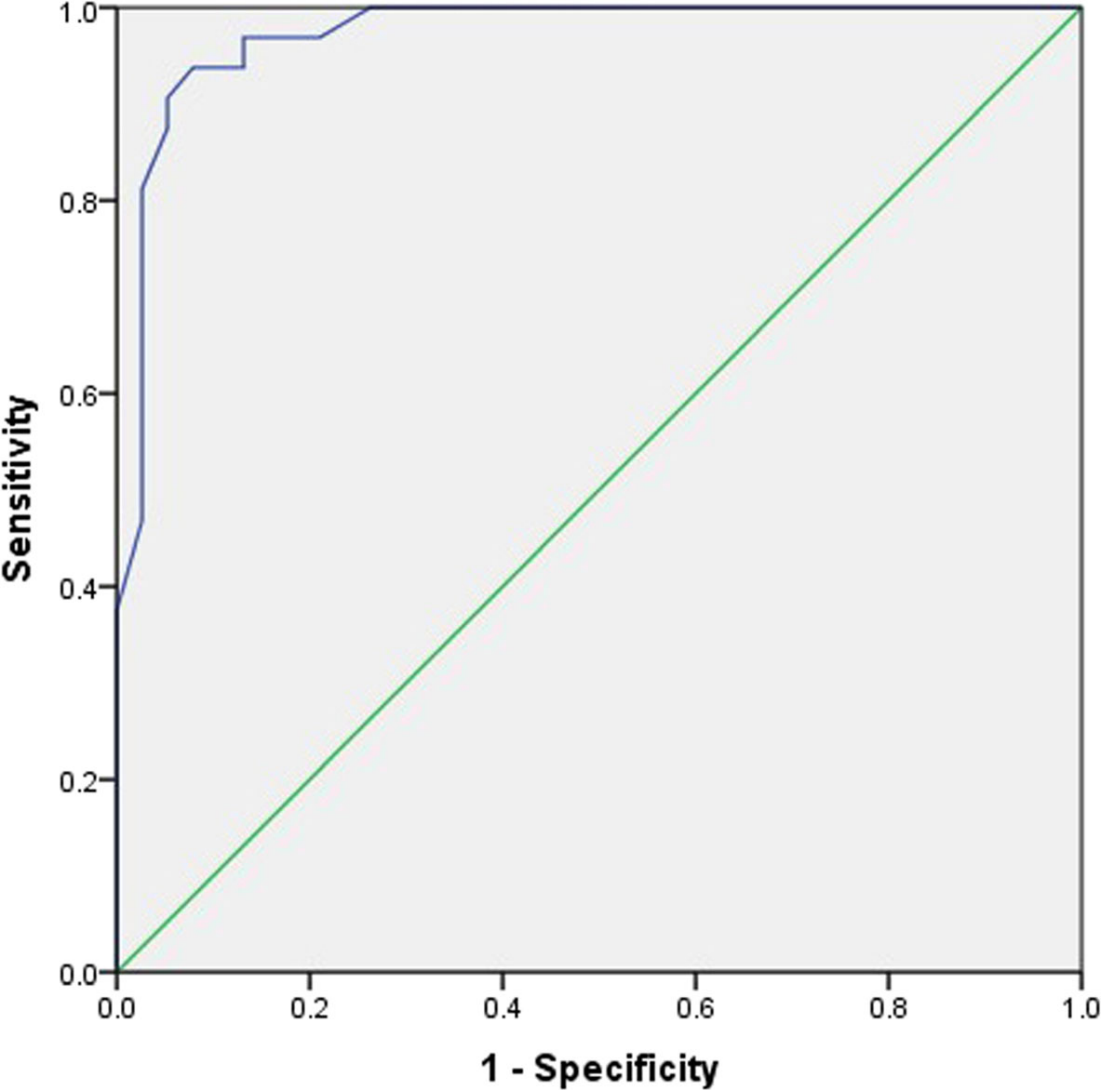
the ROC curve of APV in predicting CAD. Area under the curve (AUC) for APV: 0.972

Logistic regression analysis showed that APV was the only significant predictor of occurrence of CAD (*P* < 0.001, Table 3). Multivariate regression analysis showed that APV (*P* < 0.001) can significantly predict type of CAD involvement (Normal versus one-vessel disease, two-vessel disease or three vessel disease) (Table 4). Based on our results, APV value less than 56 cm/sec could be used in predicting CAD with a sensitivity and specificity of 96.9 and 78.9% (*P* < 0.001, AUC = 0.972), respectively (Fig. 3).

**Table 3:** Logistic regression analysis

Characteristics	B	S.E.	Wald	Sig.	Exp(B)	95% CI	
						Lower	Upper
SBP	−0.006	0.058	0.010	0.922	0.994	0.888	1.114
PP	−0.021	0.153	0.020	0.889	0.979	0.726	1.320
AS	−0.240	0.872	0.075	0.784	0.787	0.142	4.349
AD	−1.048	2.133	0.241	0.623	0.351	0.005	22.945
APV	−0.252	0.085	8.787	< 0.001	0.777	0.658	0.918

*SBP* Systolic blood pressure; *PP* Pulse pressure; *AS* Aortic stiffness; *AD* Aortic distentibility; *APV* Aortic propagation velocity

**Table 4:** Multivariate regression analysis

Characteristics	Unstandardized Coefficients		Standardized Coefficients	t	Sig.
	B	Std. Error	Beta		
SBP	−0.003	0.003	−0.104	− 0.946	0.348
PP	0.002	0.005	0.064	0.414	0.680
FH	0.073	0.076	0.066	0.970	0.336
AS	−0.012	0.019	−0.105	−0.630	0.531
AD	−0.046	0.042	−0.210	−1.100	0.276
APV	−0.019	0.002	−0.659	−8.097	< 0.0001

*SBP* Systolic blood pressure; *PP* Pulse pressure; *AS* Aortic stiffness; *AD* Aortic distentibility; *FH* Familial history; *APV* Aortic propagation velocity

## Discussion

Inflexibility of arterial wall is a marker of vascular aging and a substantial risk factor in progression of atherosclerosis and related cardiovascular disorders. Moreover, it has been mentioned before that this index is related to cardiovascular risk factors, such as smoking, hypertension, obesity, diabetes mellitus, lipid profile dysfunction and higher age [10, 15, 16]. Arterial stiffness is determined by structural and functional constituents of elastic features and influences blood pressure. This study evaluated the predictive value of APV and other arterial stiffness factors in patients with CAD and compared the results with non-CAD patients. To the best of the author’s knowledge, there are limited studies in this field and there is no study in Iran that has assessed the SYNTAX score and arterial stiffness.

In the current study, the researchers determined that APV is significantly lower in the CAD group (48.63 ± 10.31 cm/s) compared with the non-CAD group (77.75 ± 9.97 cm/s). This is similar to other studies. Yildrim et al. also showed that APV level in patients with severe risk factors in the past 10 years (37 ± 13 cm/s) was lower than patients with moderate or low risk factors [17]. Gunes et al. showed lower APV in CAD (29.9 ± 8.1 cm/s) in comparison with the normal coronary group (47.5 ± 16.8 cm/s) [18]. This indicates that decreasing APV is related to risk factors and chronicity, similar to the results of the present study. Sen et al. also demonstrated lower levels of APV in CAD (39.2 ± 13.9 cm/s) in comparison with non-CAD subjects (81.4 ± 21.4 cm/s) [12]. Chetty et al.’s study was also in accordance with the current results and showed a significant decrease of APV in CAD (41.65 ± 4.94 cm/s) rather than non-CAD (49.72 ± 6.38 cm/s) group [19]. These findings support the hypothesis that APV has a role in atherosclerotic events and arterial stiffness. It is important to note that although the ‘flow propagation of velocity’ is not a real calculation of fluid propagation between base and apex, there is a relationship with maximal detected velocity points. These results may be representative of various fluid components, which can concurrently be stimulated by local pressure gradient at various depths in front of the inflow tract [20]. As arterial stiffness is increased, APV will be decreased and this can be an early index in symptomless arterial stiffness.

Another major finding was the inverse correlation between severity of CAD (by SYNTAX score) and APV. This has not been extensively evaluated by previous studies. Only one study showed that APV has a strong and inverse correlation with the SYNTAX score [19]. Other studies used the Gensini score for severity of CAD. However, SYNTAX score has greater agreement with the guidelines when compared with the Gensini score. Sen et al. [12] and Gunes et al. [9] showed no significant correlation between severity of CAD (by Gensini score) and APV. This correlation can be representative of the severity of CAD without any intervention and could be very helpful. However, future studies on this subject are required.

Another important finding of the current study was the predictive value of APV in CAD with high sensitivity and specificity with cutoff point of 56 cm/s. Yildirim et al. reported a cutoff value of 46.5 cm/s with sensitivity and specificity of 84 and 85% for APV [17]. Chetty et al. showed a cutoff point of 47.5 cm/s with sensitivity and specificity of 76 and 72% for APV in predicting CAD [19]. Sen et al. also showed that only APV could independently predict CAD with sensitivity and specificity of 90.5 and 92.2% and with cutoff point of 60.5 cm/s [12]. The current results and other previous studies showed a spectrum of cutoff points that are almost similar and can significantly predict the CAD.

The current researchers found that there was a positive correlation between APV and AS and APV and AD. There was a negative correlation between APV, SBP and DBP. Devici et al. reported on a significantly different AS, AD, and APV in three groups and a significant relationship between APV, AS and AD and coronary ectasia score [21]. Chetty et al. also showed an inverse relation between APV and SBP, age, duration of diabetes and low LDL, which was only significant in SBP [19]. Sen et al.’s results were in line with the current findings and showed a positive correlation between APV and AS and AD [12]. This shows the correlation of arterial stiffness parameters that were in the same direction and could influence each other.

The current investigation had some limitations. Although the authors showed a good prediction by APV for CAD, a poor view in suprasternal window in echocardiography could lead to low quality images and this can result in low reproducibility of this technique. This protocol of this study was affected by age, weight, obesity, and anatomy of aorta of patients and limited access in some cases. The authors did not evaluate other parameters, such as Carotid Intima-Media Thickness (CIMT).

## Conclusion

We showed that the novel transthoracic color M-Mode, APV can significantly predict the CAD. This method is practical echocardiographic, noninvasive, simple economical approach for detecting or screening CAD. It also may be beneficial for CAD comorbidities and risk stratification and selection of high-risk individuals for CAD. One of the major important new findings of our study was the correlation between the severity of CAD with SYNTAX score and arterial stiffness. This could help physicians to make prognosis for angiographic severity of CAD. This is the first study in Iran and second study in the world that is based on new guideline of SYNTAX score which is best validated. Large scale population, multi-centric studies are recommended to validate the applicability of the method as a screening tool.

## Abbreviations

AD: Aortic distensibility
APV: Aortic propagation velocity
AS: Aortic strain
CAD: Coronary artery disease
CIMT: Carotid intima-media thickness
eGFR: Estimated Glomerular Filtration Rate
LV: Left Ventricle
LVEF: Left ventricular ejection fraction
ROC: Receiver operating characteristic
TTE: Transthoracic echocardiography
